# What impact would reducing low-acuity attendance have on emergency department length of stay? A discrete event simulation modelling study

**DOI:** 10.1136/emermed-2023-213314

**Published:** 2023-10-31

**Authors:** Hazel Squires, Suzanne Mason, Colin O'Keeffe, Susan Croft, George Millington

**Affiliations:** 1 Sheffield Centre for Health and Related Research, The University of Sheffield, Sheffield, UK; 2 National Institute for Health and Care Excellence, London, UK

**Keywords:** Length of Stay', 'Emergency Service, Computer Simulation

## Abstract

**Background:**

Long waiting times in the ED have been shown to cause negative outcomes for patients. This study aims to assess the effect in reducing length of stay of (1) preventing low-acuity attenders from attending the ED and (2) diverting low-acuity attenders at triage to a colocated general practice (GP) service.

**Methods:**

Discrete event simulation was used to model a large urban teaching hospital in the UK, as a case study, with a colocated GP service. The Centre for Urgent and Emergency Care research database patient-level database (May 2015–April 2016), secondary literature and expert elicitation were used to inform the model. The model predicted length of stay, the percentage of patients being seen within 4 hours and the incremental cost-effectiveness of the colocated GP service.

**Results:**

The model predicted that diverting low-acuity patients to a colocated GP open 9:00 to 17:00 reduces the average time in the system for higher acuity attenders by 29 min at an estimated additional cost of £6.76 per patient on average. The percentage of higher acuity patients being seen within 4 hours increased from 61% to 67% due to the reduction in the length of stay of those who were in the ED for the longest time. However, the model is sensitive to changes in model inputs and there is uncertainty around ED activity durations, for which further primary data collection would be useful.

**Conclusion:**

Reducing the proportion of low-acuity attenders at the ED could have an impact on the time in the ED for higher acuity patients due to their use of shared resources, but is insufficient alone to meet current targets. The simulation model could be adapted for further analyses to understand which other changes would be needed to meet current government targets.

**WHAT IS ALREADY KNOWN ON THIS TOPIC**

- Long waiting times and crowding in the ED lead to negative outcomes for patients.
- The literature shows that many ED visits can be managed in non-urgent care settings.

**WHAT THIS STUDY ADDS**

- The simulation model shows that diverting low-acuity patients away from the ED can have an impact on patient length of stay, particularly for those who are in the ED for the longest time; however, this would not be sufficient alone to meet current targets.

**HOW THIS STUDY MIGHT AFFECT RESEARCH, PRACTICE OR POLICY**

- Reducing low-acuity workload in the ED could be part of a multifactorial approach to tackle the problem of crowding.
- This simulation model of the ED could be used to assess alternative options for change and adapted for use within other hospitals.

## Background

Crowding in the ED is known to be associated with worse outcomes for patients including compromised quality of care, increased readmissions, prolonged hospitalisation, low patient satisfaction, high staff workload, increased ED length of stay, increased morbidity and increased mortality.^1–3^ Over the past decade, waiting times worldwide have increased. In the UK, the percentage of patients being seen within 4 hours has decreased, with the target of 95% of patients being seen within 4 hours not being met at a national level since 2014.^4^

A substantial proportion of people arriving at the ED have non-emergency conditions.^5^ They are predominantly younger patients, for whom care could have been reasonably provided in a non-emergency care setting, followed by discharge home.^6^ Diverting or avoiding these types of attendances would potentially reduce crowding in EDs and allow scarce resources to be focused on those patients in the greatest need.

The 2015 NHS Five Year Forward Views stated that ‘every hospital must have comprehensive front-door clinical streaming by October 2017, so that A&E departments are free to care for the sickest patients, including older people’.^7^ This led to interventions being rolled out including diverting low-acuity attenders to specially formed colocated general practice (GP) surgeries, and strategies designed to prevent low-acuity attenders from attending the ED.^8 9^ However, there is a little evidence^10–12^ surrounding the effectiveness of these interventions in improving outcomes compared with care as usual within the ED, and a dearth of evidence around their cost-effectiveness.^13^ This study assessed the effect in reducing length of stay in the ED and cost-effectiveness to the NHS of (1) preventing low-acuity attenders from attending the ED and (2) diverting low-acuity attenders at triage to a colocated GP service, using a simulation model of an ED in a single large urban teaching hospital in the UK, as a case study.

## Methods

Simulation modelling provides a relatively inexpensive tool to test the impact of different options for change within the ED before making any real changes to the system. Discrete event simulation (DES) is a modelling method which can be used to follow a set of individuals within a system, where every individual can be assigned characteristics, such as a low-acuity or high-acuity attender, which steer their pathway through the model. The time taken to complete each activity within the system (eg, triage or taking a blood test) can be incorporated in the model; as this can vary between patients, the times are drawn from a distribution. Resources, including different types of staff and cubicles, can also be incorporated within the model such that patients cannot be treated if the appropriate resources are unavailable, at which point queues will build up within the system. The DES can capture variation in patient arrival times, investigation and treatment times, staffing levels and processes according to time of day to reflect actual practice. The DES can then be used to assess the impact of changes.

### Understanding the problem

An understanding of this complex system and potential options for change was developed iteratively based on regular meetings and document reviews between the modelling team and the emergency medicine consultants, literature searching and a visit to observe the ED, during which input was gained from reception, triage and other nursing staff and consultants. This helped to understand how any changes in practice would fit within the broader system and which factors were particularly important in determining costs and outcomes associated with the interventions of interest. The complexity of the problem described by our clinical experts is shown in online supplemental figure A.

10.1136/emermed-2023-213314.supp1 Supplementary data

### Model boundary

The model scope was agreed between the modelling team and the emergency medicine consultants involved in the project. The model population is patients attending the ED within a single, large urban teaching hospital in the UK. The interventions assessed were: (1) low-acuity attenders redirected to a colocated GP service (a) at all times; (b) at specific times of the day and (2) fewer low-acuity attenders visit the ED because healthcare practitioners or the public are educated in making decisions about whether to attend the ED or the alternative services available, and there are accessible GP appointments. These options were compared with low-acuity attenders being triaged and investigated routinely by ED staff in the absence of these interventions. The additional costs of the colocated GP service are included. Outcomes included were length of stay, the percentage of patients being seen within 4 hours and incremental cost-effectiveness of the colocated GP service. Since reducing the proportion of low-acuity attenders may appear to increase mean length of stay because low-acuity attenders have shorter average time in the system, the impact of removal/ redirecting low-acuity attenders was assessed in terms of all attenders and higher acuity attenders.

### Model structure

**Figure 1:**
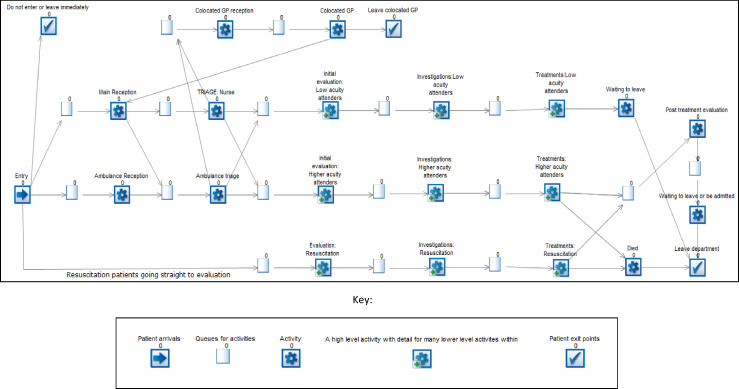
Model schematic. GP, general practice.

An overview schematic of the model developed in Simul8 software is shown in figure 1. Cog icons represent each set of key activities within the ED, while the empty squares represent queues. When the model is run, if activities are busy and there are insufficient resources (either cubicles or staff) to process the next patient, these queues are shown to build up. Patients will then move from the queue to the activity when there are sufficient resources to process them. Patients arrive at the ED either as a walk-in or via ambulance, go to reception and triage and then they may be evaluated, investigated and treated as appropriate, before they leave the ED. Patients requiring immediate resuscitation are directed straight to evaluation, bypassing reception and triage. Within each of the ‘investigation’ and ‘treatment’ cogs shown in figure 1, there is detail about which investigations and treatments each patient receives.

### Key model assumptions and parameters

#### Patient arrivals, departures, investigations and treatments

We used routinely collected patient data held in the University of Sheffield Centre for Urgent and Emergency Care research database (CUREd),^14^ which includes record-level patient data from the Yorkshire and Humber region ED attendances. We used the following data from ED attendances from the large urban hospital in the region: arrival time and date, arrival mode, coded investigations and treatments, resuscitation cases, departure method, acuity, time to end treatment and departure time and date. Data from 1 May 2015 to 30 April 2016 was used to model the ED without a colocated GP service, since a limited colocated out of hours GP service was implemented in May 2016. Minor data cleaning was undertaken in the statistical software package R. Based on the dataset, 20% of attendances were defined as low-acuity and could potentially be seen elsewhere than the ED. There is no universally agreed definition of low-acuity attenders, but this study used the criteria previously set out by O'Keeffe *et al*,^15^ that low-acuity attenders receive no investigations or treatments except urine tests, advice and prescriptions and must be discharged without follow-up or with referral to a GP. This is a conservative definition to avoid overestimating the number of low-acuity attenders. We defined all the remaining patients as ‘higher acuity’ since some of these will not be urgent attenders.

All arrival times, investigations and treatments in the model were based on data for the hospital ED. To calculate the time taken for patients to leave the ED following treatment, we needed the departure time (the time patients left the ED) and conclusion time (the earliest of the time the patient left the ED and the time treatment in the ED was completed). However, there were no departure times recorded for the hospital ED being modelled, so the average departure times for all of the hospital ED data within the CUREd dataset were used to estimate this.

#### Activity durations

The electronic data collection system used by the hospital was used to inform the duration of triage consultation by arrival mode (ambulance or walk in). A probability density function was provided, to which a gamma distribution was shown to be the best fit, since the data was skewed. This was assumed to be the same whether or not patients were referred to a colocated GP service as a result of triage. No data was identified within the dataset or within any secondary literature sources which provided detailed information about the duration of other activities within the ED at the same granular level. Thus, the remaining activity durations were based on an elicitation exercise. Experts were asked a series of questions via an online application to estimate the time taken to complete an activity, and the results of this were then verified by each expert. Two emergency medicine consultants, one senior nurse and one middle grade doctor, completed the exercise, which was then used to quantify the mean and variability of the time taken to complete each activity in the model. The patient was assumed to be evaluated by a clinician two times; once for all patients before any investigations or treatments and once after treatment if received. In practice, some patients may receive more than two evaluations; however, the intention was to capture the total amount of time for evaluation, without describing the detail. The key outputs of the elicitation exercise are shown in online supplemental table A.

#### Staffing and cubicle levels

In 2017, the deputy operations director for emergency and acute medicine at the same large urban hospital completed a survey providing data on the number of cubicles and the number of each staff type working within the ED at any one time (see online supplemental table B). It was assumed that this was consistent with cubicle and staffing levels when our data was collected in 2015–2016. For the duration of all activities, at least one member of staff and a cubicle were assumed to be required. There were assumed to be separate cubicles for resuscitation patients and patients classed as majors/minors. It was not possible to distinguish between patients classed as major and minor in the model because this information was not recorded within the dataset; however, clinical input suggested that while cubicles are assigned for majors or minors, in practice they would be used for either group as needed. Two emergency medicine consultants identified which staff types would undertake each activity (online supplemental table C). It was assumed in the model that the most junior relevant staff type(s) available would undertake the activity. To account for breaks, it was assumed that staff would only be available for 89% of their working hours, based on an unpublished nursing staffing tool.^16^ Patients queuing for the same activity were prioritised by entry mode (ambulance, then walk in), followed by wait time. Staff prioritised specific investigations or treatments if higher acuity patients had been waiting 60 minutes or low acuity patients had been waiting 120 minutes.

#### Costs

Only the additional costs of the colocated GP service were included, as it was assumed that costs within the ED would not be decreased due to fewer staff by reducting non-urgent attenders. For the colocated GP, the hourly cost of a GP, with nursing and administrative support, as well as qualification costs (depreciated over the expected working life of a doctor), overheads, capital costs (depreciated over 60 years at a discount rate of 3.5%), was taken from the latest Personal Social Services Research Unit (PSSRU) data.^17^ This provided an hourly cost to run the colocated GP service of £271 which was multiplied by the number of hours the GP service was open for each modelled scenario and by 28 days, which was then divided by the average number of attenders across each 28-day cycle to provide a per-patient cost.

#### Model verification and validation

Model verification checks that the model is implemented as planned. The model was verified by another modeller (GM), where all model code was checked and tested. During this process, any discrepancies between the planned model and the implemented model were corrected. The model code can be made available on request to the authors.

**Figure 2:**
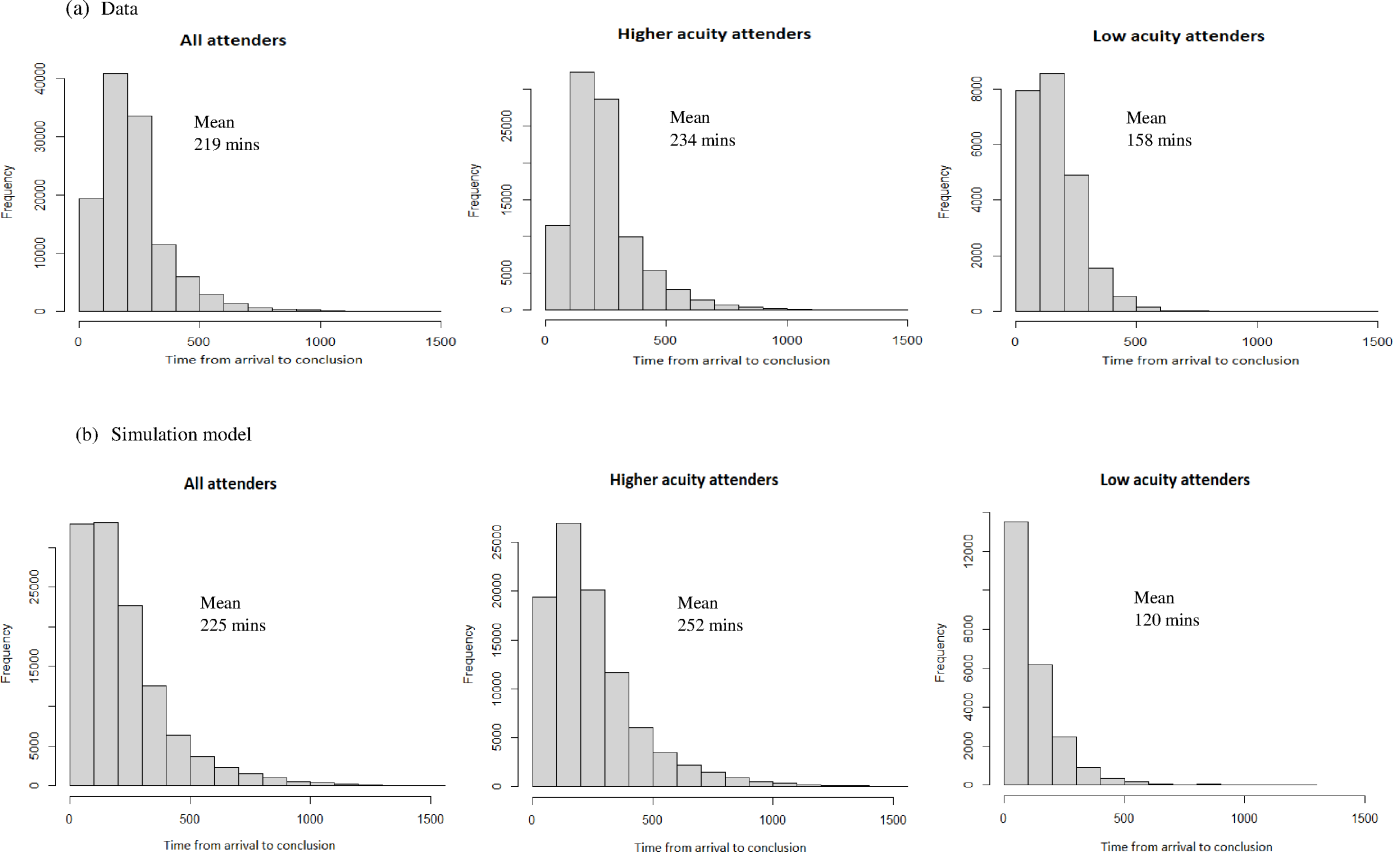
Comparison of simulation model and dataset outputs. Histograms of the time from arrival to conclusion for (A) the data set and (B) the simulation model output, for all attenders, higher acuity attenders and low-acuity attenders. For example, the first graph shows that within the data set for all attenders being seen within a 28-day period, just under 2000 patients took less than 100 min to leave the ED, with the large majority being discharged within 500 min and the mean time within the ED being 219 min.

Model validation checks that the model reasonably represents the system it is supposed to. The assumptions and parameters within the model were validated by an emergency medicine consultant throughout model development (SMM and SC). An emergency medicine consultant also validated the results of the elicitation exercise (SC). The mean and distribution of length of stay within the ED was compared between the simulation and the CUREd dataset. Analyses of alternative options were not undertaken until the simulation model reasonably represented the current system. The outputs of this validation exercise, in terms of length of stay, are shown in figure 2 below. This shows that the model represents the current system reasonably well; however, low-acuity attenders have a lower length of stay in the simulation model than in the dataset on average. This means that the model may slightly underestimate the impacts of the interventions being assessed.

### Model analyses

Given that the ED is never empty, a ‘warm-up’ period was run, within which time results are not collected, to allow patients to enter the simulation and build up to represent the current system. The warm-up period was estimated using Welch’s graphical approach,^18^ which involves running the model for an increasing period of time until the outputs are relatively stable. The model had reached a steady state by 1 week (online supplemental figure B); hence a ‘warm-up’ period of 1 week was used. The results of the model were collected over a 4-week period. The model was run 12 times to allow for monthly variation of interarrival times and the number of investigations and treatments required, based on the variation in the CUREd dataset. The variability in activity durations was incorporated using different random number seeds during each model run.

For the alternative options, a proportion of patients were either (1) removed from the ED before using any resources; or (2) diverted to the colocated GP from triage which could be open (a) at all times; (b) 9: 00 to 17:00 or (c) 9:00 to 21:00. These times were chosen because the dataset showed most patients arriving between 9:00 and 17:00, with a large proportion also arriving between 17:00 and 21:00. Overall, 1% of patients were assumed to be referred back to the ED following GP attendance.

## Results

The length of stay within the ED from each intervention assessed within the model for all attenders is shown in table 1 and for higher acuity attenders is shown in table 2. The model predicted that diverting low-acuity patients to a colocated GP open 9:00 to 17:00 reduces the average time in the system for higher acuity attenders by 29 min. There are smaller changes in the median length of stay than the mean because reducing low-acuity attenders leads to a reduction in the length of stay of those who were in the ED for the longest time period. Thus, the percentage of patients being seen within 4 hours increases, with a greater percentage increase for higher acuity attenders. The distributions of time spent in the ED are shown in online supplemental figure C. The diversion to colocated GP interventions appears slightly less effective than the equivalent removal interventions; however, the former may be a more practical solution.

**Table 1:** Length of stay for all attenders for each intervention

Model	Mean (SD) (min)	Median (min)	IQR range (min)	% <4 hours
Usual care	214 (173)	170	92–284	66.8%
33% removal	193 (141)	160	88–261	70.7%
66% removal	185 (125)	157	89–252	72.5%
100% removal	185 (116)	159	93–249	72.8%
33% redirected to GP, open at all times	199 (147)	164	90–268	69.4%
66% redirected to GP, open at all times	189 (129)	160	91–255	71.9%
100% redirected to GP, open at all times	189 (119)	162	96–254	71.9%
100% redirected to GP, open 9:00–21:00	196 (135)	164	94–262	70.4%
100% redirected to GP, open 9:00–17:00	198 (144)	163	91–265	69.9%

GP, general practice.

**Table 2:** Length of stay for higher acuity attenders for each intervention

Model	Mean (SD) (min)	Median (min)	IQR range (min)	% <4 hours
Usual care	239 (178)	195	113–310	61.2%
33% removal	210 (142)	177	104–278	66.9%
66% removal	194 (125)	165	97–260	70.8%
100% removal	185 (116)	159	93–249	72.8%
33% redirected to GP, open at all times	215 (149)	180	106–284	65.8%
66% redirected to GP, open at all times	197 (128)	168	99–262	70.1%
100% redirected to GP, open at all times	189 (119)	162	96–254	71.9%
100% redirected to GP, open 9:00–21:00	202 (136)	170	100–269	69.0%
100% redirected to GP, open 9:00–17:00	210 (146)	174	102–277	67.4%

GP, general practice.

The full incremental cost-effectiveness analysis for the alternative colocated GP intervention opening times is shown in table 3. The incremental cost-effectiveness ratio can be interpreted as the additional cost (£) per minute saved in the system by the intervention compared with the next best alternative for higher acuity attenders. For example, opening the colocated GP from 9:00–17:00 would result in an estimated 29 min shorter length of stay for higher acuity attenders on average than caring for all patients in the ED, at an extra cost of £6.76 per patient in the system, or 23 pence for every minute saved. However, the incremental cost increases if the colocated GP service is open all hours, the cost rises to 78 pence per minute saved because there is less benefit of referring patients when the ED is less crowded.

**Table 3:** Fully incremental cost-effectiveness analysis of mean time in the system

Model	Mean time in the system (min)	Costs per patient (£)	Incremental effectiveness (min)	Incremental costs	Incremental cost-effectiveness ratio
Usual care	239	0	N/A	N/A	N/A
GP open 9:00–17:00	210	6.76	29	6.76	0.23
GP open 9:00–21:00	202	10.14	8	3.38	0.42
GP always open	189	20.28	13	10.14	0.78

GP, general practice.

For comparison with other interventions funded by the NHS, at a willingness to pay of £20 000 per quality-adjusted life year gained, for the most cost-effective option of colocated GP open 9:00–17:00, 1 QALY would need to be saved per 2958 patients being in the ED for an average reduction in length of stay of 29 min.

## Discussion

This study suggests that reducing the number of low-acuity attenders to the ED has the potential to improve flow and reduce waiting times for all patients attending, including higher acuity patients. All outcomes for each scenario tested reducing the low-acuity workload were improved over the usual care model. Both the main analysis and the cost-effectiveness analysis implied diminishing returns from reducing greater proportions of low-acuity attenders in the ED. This is in part because for some investigations and treatments multiple types of staff (eg, consultant and nurse) are required simultaneously and when both staff types are very busy due to crowding it is less likely that they will both be available at once, hence slowing the system down further. Opening the GP service at times with more activity appears to be more effective than percentage-based diversion. Since lower acuity attenders spent less time in the model than in the dataset, it is possible that these strategies may have a larger impact in practice.

Other published studies have used simulation to assess the impact of alternative service configurations for the ED^19^; however, none of them have considered the impact of preventing attendance by or removing low-acuity attenders. Ferreira *et al* 20 developed a simulation model of a Brazilian ED, which suggested that the interarrival time and triage capacity were of key importance to patient flow, with length of stay increasing substantially above a certain threshold. This is consistent with the results of our simulation study.

### Implications for policy

There is a lack of evidence that reducing ED processing of low-acuity patients can impact on the care of sicker patients.^21 22^ Our study shows that if streaming of low-acuity attenders is implemented successfully, there are potential benefits for sicker patients in the ED, which is an important factor when considering access or streaming approaches. Within our study, low-acuity attenders were identified retrospectively from routine data. In practice, tools will be needed to identify these patients prospectively.

Our model can also help with policy decisions such as the opening times of colocated GP services. Evidence about the effectiveness of interventions to reduce low-acuity attenders in the ED is mixed,^23–25^ and effectiveness may be influenced by local context and adoption.^26^ Further research is required about the most effective strategies for reducing low-acuity attenders to inform future policy decisions. A multifaceted approach should be considered so that the numbers attending the ED do not continue to rise.

### Study limitations and further research

The simulation model was developed based on one urban teaching hospital in the UK, as a case study. There is variation in the way in which EDs are organised and run. It would be beneficial to develop modified versions of the model for other localities with different key features, for example, smaller hospitals and those with different staffing arrangements. The current model only considers interventions to reduce low-acuity attenders; it could also be used to test alternative options for change within the ED that may reduce crowding and improve flow, including improving patient discharge from the ED.

There are some limitations with the data used by the model, including that: (1) there were some missing data which needed to be imputed; (2) there was no data around whether patients were classed as ‘majors’ or ‘minors’; (3) there was no data for departure times for the hospital ED being modelled; and (4) there was no data around the proportion of patients who would be re-referred to the ED after being seen by the colocated GP. It is, however, expected that the assumptions made regarding these would not have a substantial impact on model results. In addition, the data used within the simulation is from 2015 to 2016, and there is evidence that EDs have become more crowded since then,^4^ thus removing low-acuity attenders may have a greater impact on length of stay than predicted here. The model does not account for potential changes in the types of patients who attend the ED since the COVID-19 pandemic.

Other than triage, there was no data around the distribution of time taken for investigations and treatments. This is particularly important because the model is highly sensitive to small changes in assumptions and parameters. Further primary research to understand how long procedures take within the ED, including the variability within and between hospitals, could provide less uncertainty for future modelling. In addition, further primary research could derive utility values or specific mortality data related to waiting times, which would enable cost-utility analysis so that the value of opening the colocated GP can be compared with other healthcare interventions outside of the ED.

In simulating the ED, it became apparent just how complex the ED department is, and how important it is to understand the nuances of the department to be able to consider potential improvements. The validation exercise showed that the model underestimates the length of stay for low-acuity attenders and this is due to the model’s simplifying assumptions. The decision-making behaviour of clinical experts working in the ED was found to adapt according to the specific circumstances within the ED at that time, as well as varying between individual clinical experts. It is challenging to model these nuances, but it is important to include any which will substantially affect outcomes. While it is not possible (or desirable) to exactly represent reality with a simulation model, the model could be improved with a greater understanding of how staff prioritise work and move between activities within the ED. Further observational and qualitative research is needed around staff decision-making behaviour to improve future model assumptions.

## Conclusions

The simulation model suggested that reducing the number of low-acuity attenders to the ED, either by avoiding the attendance or by diverting them to a colocated GP following triage, successfully reduced the length of stay in the ED. The option which is likely to be the most cost-effective and practical is to divert low-acuity attenders to a colocated GP service from 9:00–17:00. However, additional interventions would be required to meet current government targets.

## Data Availability

Data not available due to ethical restrictions. Model code may be shared upon reasonable request.
