# Supplementary material for: What impact would reducing low-acuity attendance have on emergency department length of stay? A discrete event simulation modelling study
Source: Emerg Med J. 2023 Oct 31;41(1):27–33. doi: 10.1136/emermed-2023-213314 (PMC10804000; doi:10.1136/emermed-2023-213314)
Supplement: Supplementary data [file emermed-2023-213314supp001.pdf]

Supplementary material

Figure A: Conceptual model of the problem

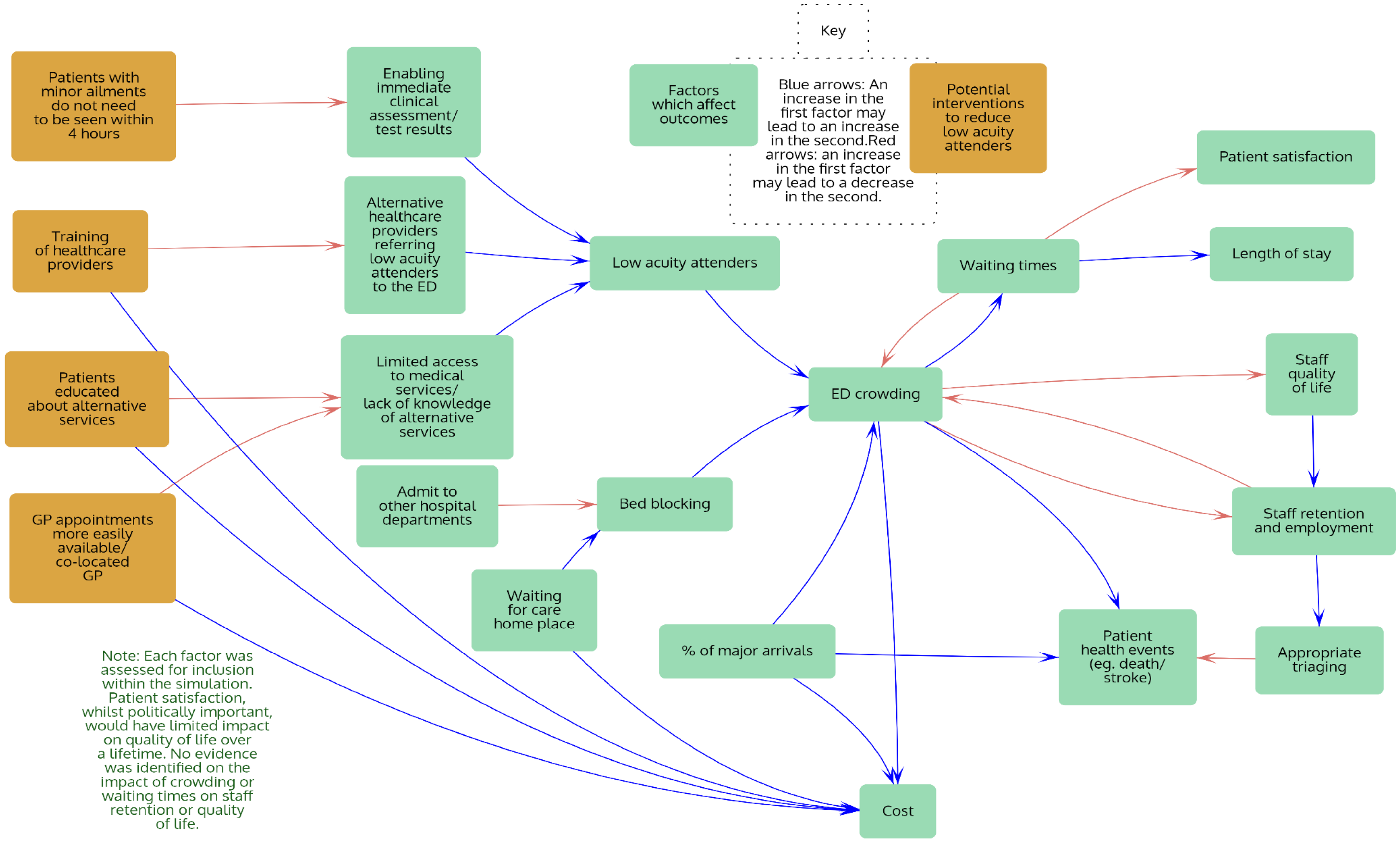

Table A: Elicitation results for time taken for each activity in the ED

Activity	Gamma -alpha	Gamma - beta	Mean
Main reception	2.25	1.33	3.0
Ambulance reception	2.25	1.33	3.0
Nurse triage	3.05	1.81	5.5
Ambulance triage	3.87	1.54	6.0
Pre-investigation	27.26	0.62	16.9
X-ray book	2.56	0.39	1.0
X-ray travel	11.11	0.45	5.0
X-ray test	2.56	7.81	20.0
X-ray results	4	0.5	2.0
Blood book	2.56	0.39	1.0
Blood perform	36.53	0.29	10.6
Blood process	5.22	11.48	59.9
Blood results	4	0.5	2.0
ECG test	8.16	0.61	5.0
ECG results	4	0.5	2.0
Urine test	16	0.31	5.0
Urine results	2.84	1.85	5.3
Other scan book	2.56	0.39	1.0
Other scan travel	11.11	0.45	5.0
Other scan test	7.11	4.22	30.0
Other scan results	2.37	2.11	5.0
Eye test	11.11	0.68	7.6
Other investigation	16	0.31	5.0
Advice	3.41	0.88	3.0
Other drugs	11.11	1.35	15.0
Prescription	5.33	1.41	7.5
Pot/splint/crutch	5.54	4.52	25.0
Wound cleaning	3.11	2.41	7.5
Sutures	12.96	1.74	22.6
Manipulation/physio	5.88	5.1	30.0

Dressing	3.11	2.41	7.5
Oxygen/nebuliser	0.4	14.04	5.6
Wound closure exc. Sutures	6.25	1.2	7.5
Bandage support	6.25	0.8	5.0
Incision/drain	25	0.5	12.5
Recording vital signs	0.4	14.04	5.6
Remove foreign body part	7.55	1.62	12.2
Lavage/emesis/charcoal/eye irrigation	7.55	1.62	12.2
Urinary catheter	18.11	0.85	15.4
Other treatment	5.67	2.08	11.8

Table B: Number of staff and cubicles at the hospital

Weekday	Consultant	Associate	Middle Grade	Junior Doctor	Senior Nurse	Junior nurse	Specialist nurse	Support worker	Receptionist	Porters
00:00	0	0	1	4	2	11	0	5	2	3
01:00	0	0	1	4	2	11	0	5	2	3
02:00	0	0	1	4	2	11	0	5	2	3
03:00	0	0	1	4	2	11	0	5	2	3
04:00	0	0	1	3	2	11	0	5	2	3
05:00	0	0	1	3	2	11	0	5	2	3
06:00	0	0	1	3	2	11	0	5	2	3
07:00	0	0	1	3	4	11	1	5	3	4
08:00	2	0	2	3	4	11	1	5	3	4
09:00	2	0	2	3	4	11	2	5	3	4
10:00	3	0	2	3	4	11	2	5	3	4
11:00	3	0	2	3	4	11	2	5	3	4
12:00	3	0	2	3	4	11	2	5	3	4

13:00	3	0	2	5	4	11	3	5	3	4
14:00	3	0	2	5	4	11	3	5	3	4
15:00	3	0	2	5	4	11	3	5	3	4
16:00	3	0	2	5	4	11	3	5	3	4
17:00	3	0	2	5	4	11	3	5	3	4
18:00	3	0	2	6	4	11	2	5	3	4
19:00	3	0	2	6	2	11	2	5	3	4
20:00	3	0	2	6	2	11	2	5	3	4
21:00	3	0	2	6	2	11	0	5	3	3
22:00	3	0	2	6	2	11	0	5	2	3
23:00	3	0	2	4	2	11	0	5	2	3

Weekend	Consultant	Associate	Middle Grade	Junior Doctor	Senior Nurse	Junior nurse	Specialist nurse	Support worker	Receptionist	Porters
00:00	0	0	2	4	2	11	0	5	2	3
01:00	0	0	2	4	2	11	0	5	2	3
02:00	0	0	1	4	2	11	0	5	2	3
03:00	0	0	1	4	2	11	0	5	2	3
04:00	0	0	1	3	2	11	0	5	2	3
05:00	0	0	1	3	2	11	0	5	2	3
06:00	0	0	1	3	2	11	0	5	2	3
07:00	0	0	1	3	4	11	1	5	3	4
08:00	1	0	1	3	4	11	1	5	3	4
09:00	1	0	1	3	4	11	2	5	3	4
10:00	2	0	1	3	4	11	2	5	3	4
11:00	2	0	1	3	4	11	2	5	3	4
12:00	2	0	1	3	4	11	2	5	3	4

13:00	2	0	1	5	4	11	3	5	3	4
14:00	2	0	1	5	4	11	3	5	3	4
15:00	2	0	1	5	4	11	3	5	3	4
16:00	2	0	2	5	4	11	3	5	3	4
17:00	2	0	2	5	4	11	3	5	3	4
18:00	2	0	1	6	4	11	2	5	3	4
19:00	2	0	1	6	2	11	2	5	3	4
20:00	1	0	1	6	2	11	2	5	3	4
21:00	1	0	1	6	2	11	0	5	3	3
22:00	1	0	2	6	2	11	0	5	2	3
23:00	1	0	2	4	2	11	0	5	2	3

Table C: Staff allocation of activities

Activity	Staff type								
	**Consultant**	**Associate Specialist doctor**	**Middle grade doctor**	**Junior doctor**	**Senior nurse**	**Specialist nurse**	**Junior nurse**	**Support worker/ technician**	**Porter**
Reception	-	-	-	-	-	-	-	-	-
Walk in triage	-	-	-	-	Performs	-	-	-	-
Ambulance triage	Performs	-	-	-	Performs	-	-	-	-
X-ray	Interprets x-ray result	Interprets x-ray result	Interprets x-ray result	Interprets x-ray result	-	Interprets x-ray result	-	-	Takes patient for x-ray
Blood	Interprets results	Interprets results	Interprets results	Interprets results	-	Interprets results	-	Takes blood	-
ECG	Interprets results	Interprets results	Interprets results	Interprets results	-	Interprets results	-	Performs ECG	-
Urine inc pregnancy	Interprets results	Interprets results	Interprets results	Interprets results	-	Interprets results	Collects urine from patient and dips	Collects urine from patient and dips	-
Other scan	Interprets results	Interprets results	Interprets results	Interprets results	-	Interprets results	-	-	Takes patient for scan

Eye test	Performs test	Performs test	Performs test	Performs test	-	Performs test	-	-	-
Observations	Interprets results	Interprets results	Interprets results	Interprets results	Performs	Interprets results	Performs	Performs	-
Advice	Provides	Provides	Provides	Provides	Provides	Provides	Provides	x	-
Prescription	Prescribes	Prescribes	Prescribes	Prescribes	Administers	Prescribes	Administers	-	-
Other drugs	Prescribes	Prescribes	Prescribes	Prescribes	Administers	Prescribes	Administers	-	-
Pot/Splint/crutches	-	-	-	-	Performs and provides	Performs and provides	Performs and provides	Performs and provides	-
Wound cleaning	-	-	-	-	Performs	Performs	Performs	Performs	-
Sutures	Performs	Performs	Performs	Performs	-	Performs	-	-	-
Manipulation/physio - physio	Arranges	Arranges	Arranges	Arranges	-	Arranges	-	-	-
Manipulation/physio - manipulation	Performs	Performs	Performs	-	-	Performs	-	-	-
Dressing	-	-	-	-	Applies dressing	-	Applies dressing	Applies dressing	-
O-ygen/nebuliser	Prescribes	Prescribes	Prescribes	Prescribes	Administers	Prescribes	Administers	-	-
Wound Closure E-cluding suture	-	-	-	-	Applies steristrips/glue	-	Applies steristrips/glue	Applies steristrips/glue	-

Bandage/support	-	-	-	-	Applies bandage/splint	-	Applies bandage/splint	Applies bandage/splint	-
Incision/drain	Performs	Performs	Performs	-	-	-	-	-	-
Recording vital signs	-	-	-	-	Performs vital signs	-	Performs vital signs	Performs vital signs	-
Removal of a foreign body	Performs	Performs	Performs	Performs	-	Performs	-	-	-
Lavage/emesis/charcoal/eye irrigation	-	-	-	-	Performs	-	Performs	-	-
Inserting urinary catheter	-	-	-	Inserts	Inserts	Inserts	Inserts	-	-
Defibrillation	Performs	Performs	Performs	Performs	-	Performs	-	-	-
Resuscitation	Performs	Performs	Performs	Performs	Performs	Performs	Performs	Performs	-
Minor surgery	Performs	Performs	Performs	Performs with support	-	Performs	-	-	-
Giving anaesthesia - local	Performs	Performs	Performs	Performs	-	Performs	-	-	-
Giving anaesthesia - blocks and sedation	Performs	Performs	Performs	-	-	-	-	-	-
X-ray review	Performs	Performs	Performs	Performs	-	Performs	-	-	-
Epistaxis control	Cauterises	Cauterises	Cauterises	Cauterises	-	Cauterises	-	-	-
Providing supplemental o-ygen	Prescribes	Prescribes	Prescribes	Prescribes	Administers	Prescribes	Administers	-	-

Continuous positive airways pressure/nasal intermittent positive pressure ventilation/bag valve mask	Orders	Orders	Orders	-	-	Administers	Administers	-	-
Inserting arterial line	Performs	Performs	Performs	-	-	-	-	-	-
Active rewarming of the hypothermic patient	-	-	-	-	Administers	-	Administers	Administers	-
Occupational therapy	-	-	-	-	Arranges	-	Arranges	-	-
Social work intervention	-	-	-	-	Arranges	-	Arranges	-	-
Initial evaluation	Performs	Performs	Performs	Performs	-	Performs	-	-	-
Post treatment evaluation	Performs	Performs	Performs	Performs	Supports	Performs	Supports	-	-

Note: Receptionist works on reception

Figure B: Estimating an appropriate warm up period

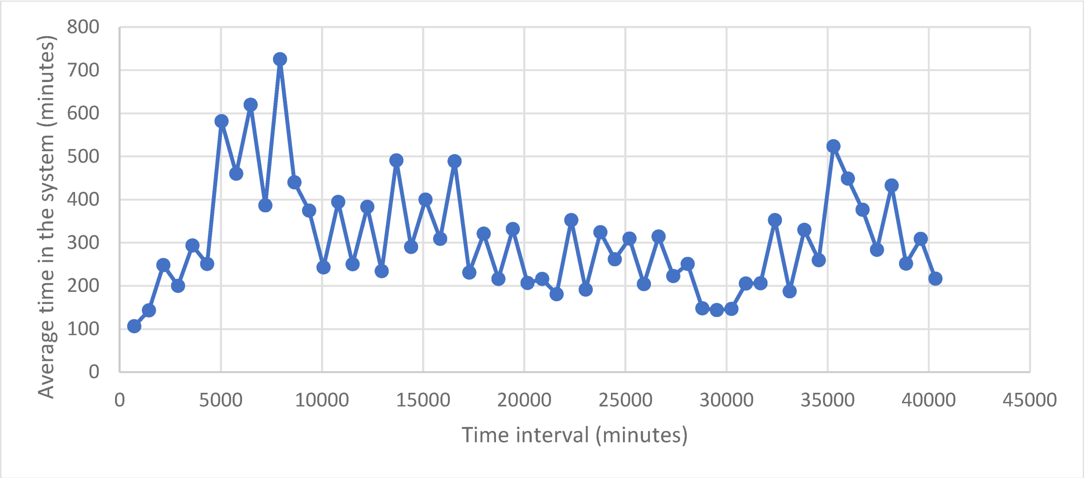

Figure C: Histograms of time in the system from the model

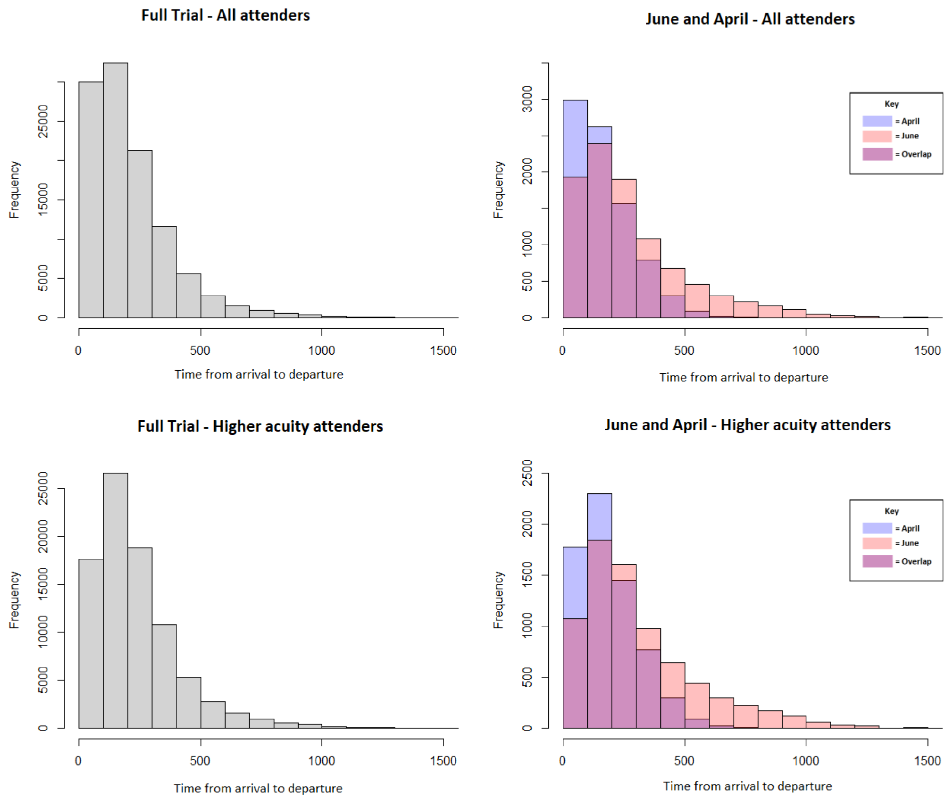

Histograms of time in the system from the usual care model

Note: The month with the highest number of attendances was June (9,407 patients) and the month with the lowest attendances was April (8,396 patients).

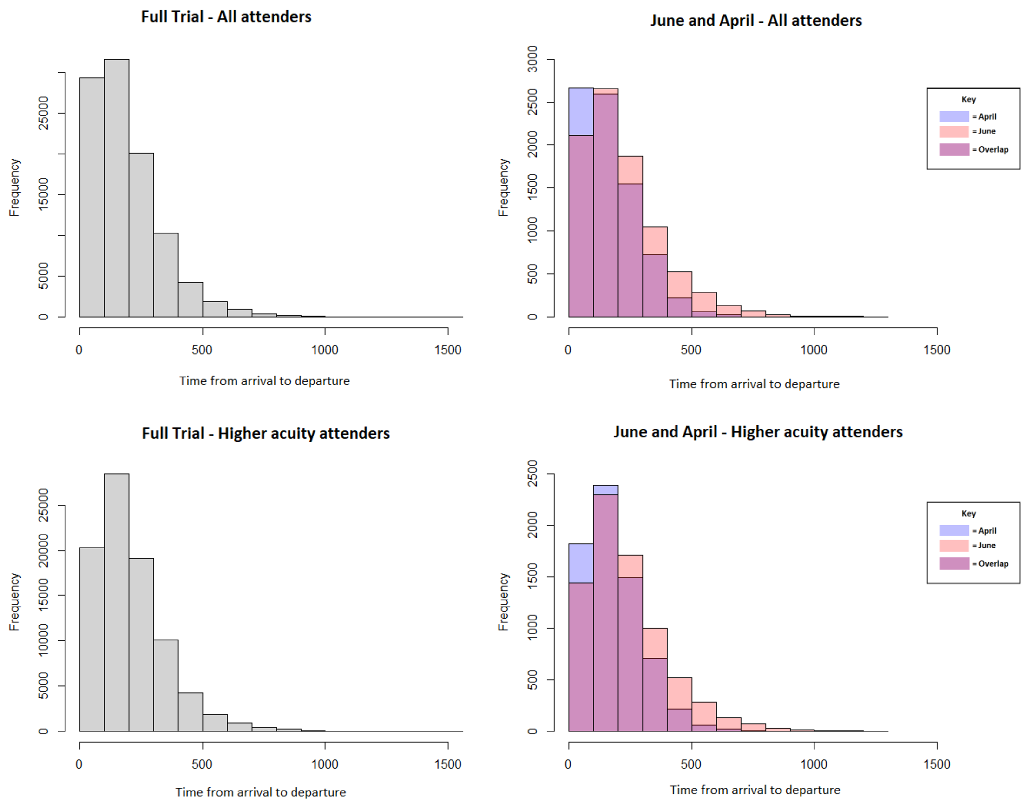

Histograms of time in the system from the model that removes 33% of low acuity attenders.

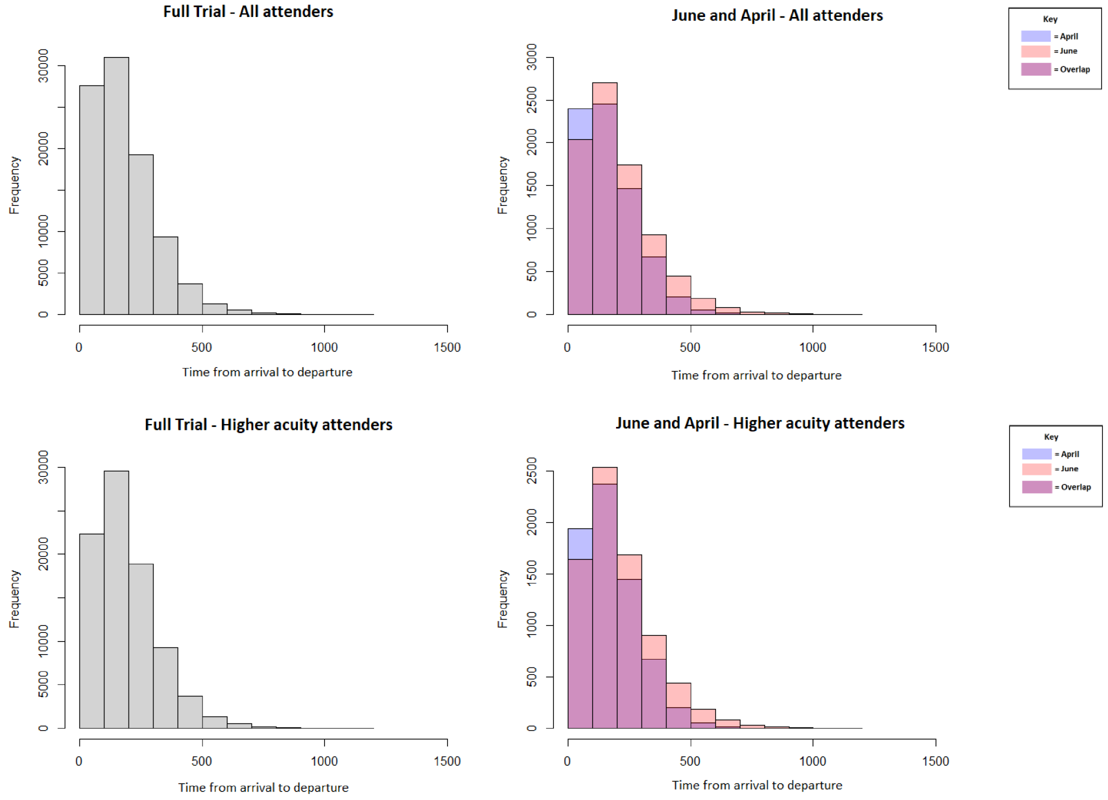

Histograms of time in the system from the model that removes 66% of low acuity attenders.

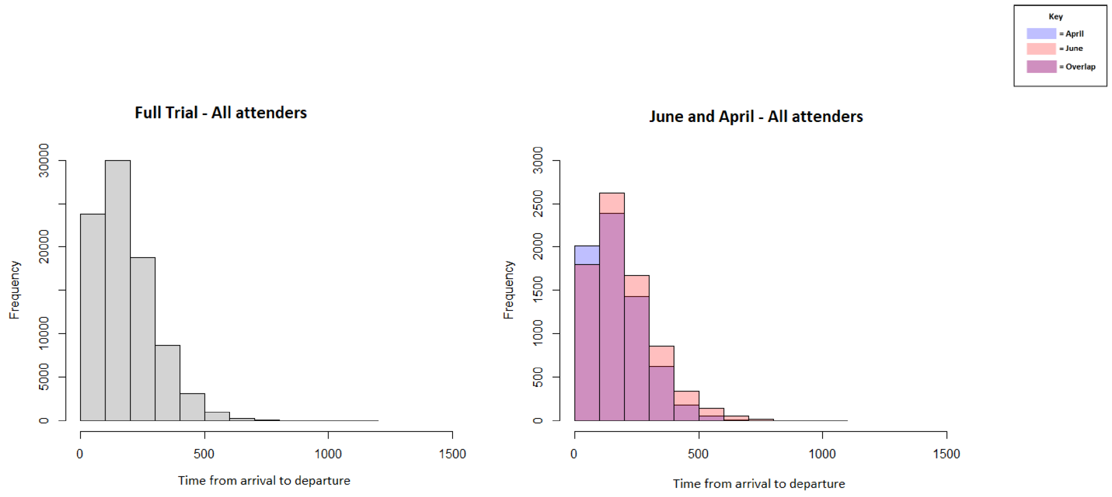

Histograms of time in the system from the model that removes 100% of low acuity attenders. As only higher acuity attenders remained in the system, only one set of graphs is presented.

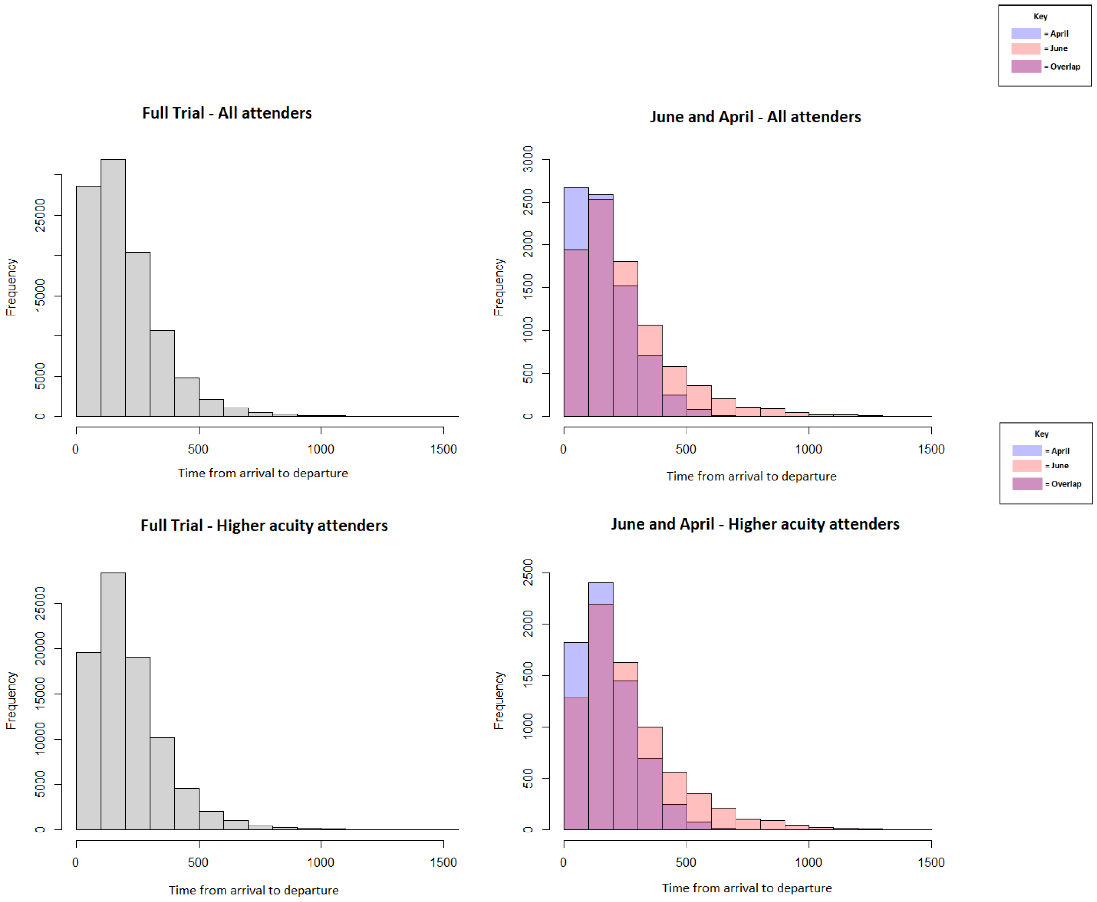

Histograms of time in the system from the model that diverts 33% of low acuity attenders to a GP service.

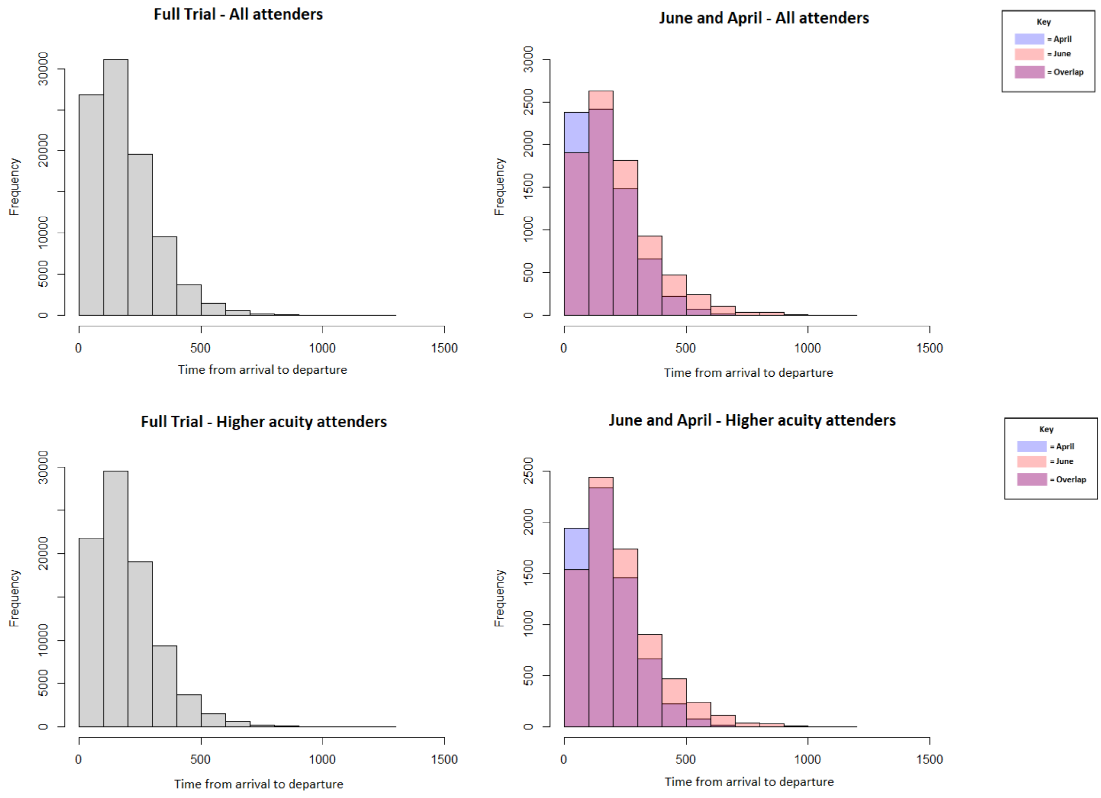

Histograms of time in the system from the model that diverts 66% of low acuity attenders to a GP service.

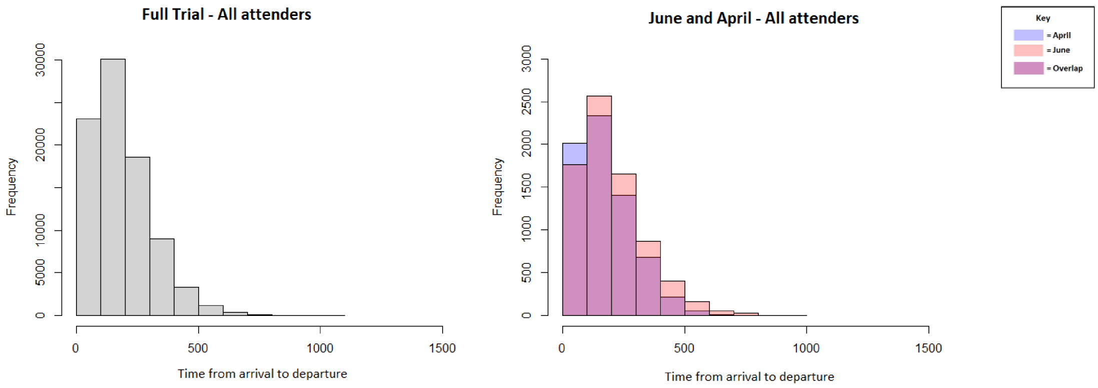

Histograms of time in the system from the model that diverts 100% of low acuity attenders to a GP service. As only higher acuity attenders completed the system, only one set of graphs is presented.

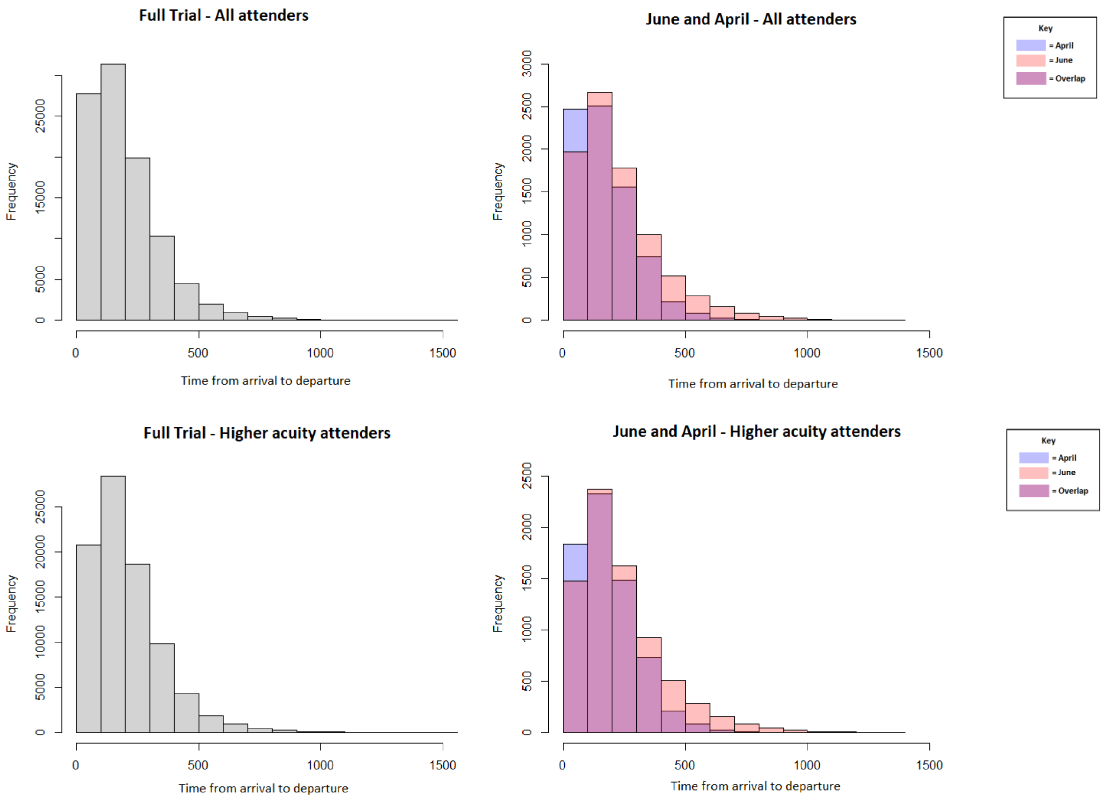

Histograms of time in the system from the model that diverts low acuity attenders to a GP service between 9am and 5pm.

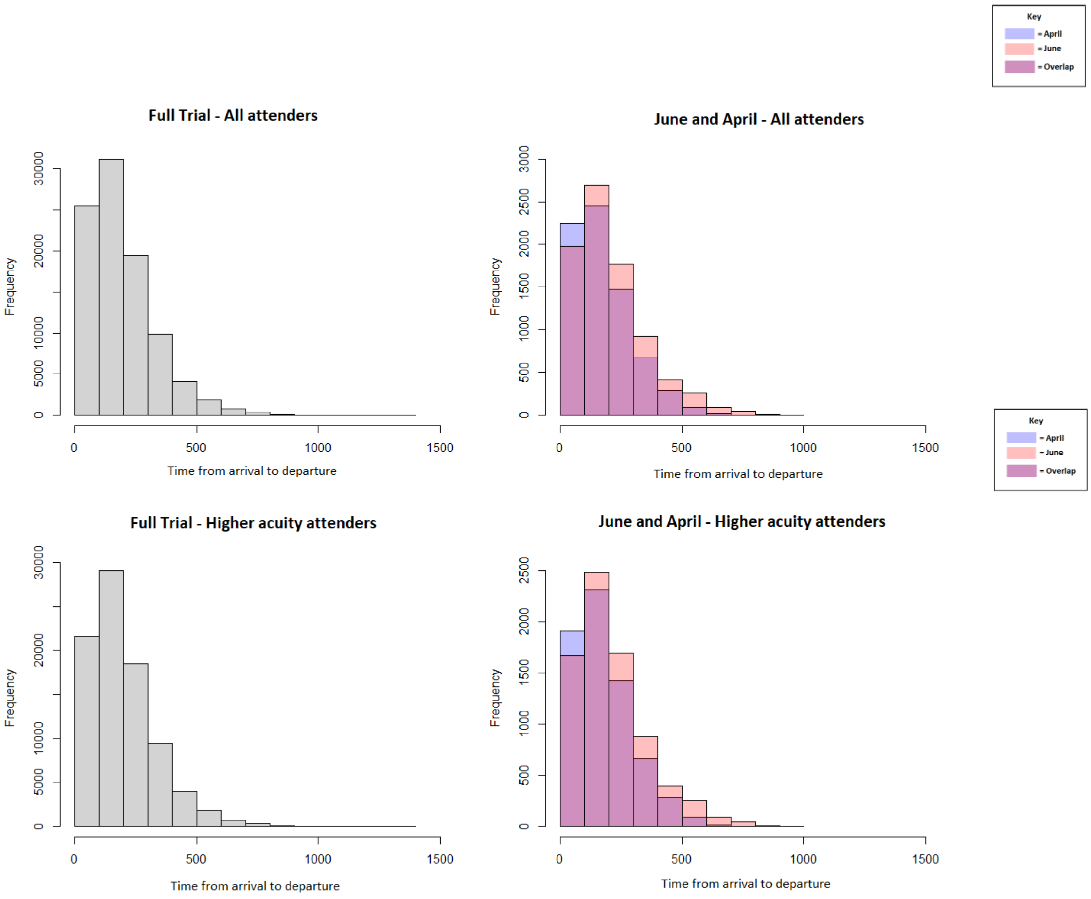

Histograms of time in the system from the model that diverts low acuity attenders to a GP service between 9am and 9pm.
